# Reviewer social class influences responses to online evaluations of an organization

**DOI:** 10.1371/journal.pone.0205721

**Published:** 2018-10-18

**Authors:** Suzanne Horwitz, Balázs Kovács

**Affiliations:** Yale University School of Management, New Haven, United States of America; Saint Peter's University, UNITED STATES

## Abstract

This paper examines social class-based differences in influence in online review contexts. We explore four mechanisms for how a review writer’s social class may affect readers’ evaluations of the organization. First, we argue that, via a “contagion” process, organizations reviewed by higher-class individuals will be evaluated more positively than organizations reviewed by lower-class individuals. Second, we expect that higher-class reviewers will be seen as more knowledgeable; thus, their opinions will be more influential in shaping others’ opinions. Third, we expect that reviewers will be seen more influential when they review organizations that match their social class. Fourth, we expect people to be more influenced by those who share their own class background. A large-scale observational study of reviews (N = 1,234,665) from Yelp.com finds support for the contagion, the organization-reviewer social class matching, and the reviewer-participant social matching hypotheses, but disconfirms the hypothesis that higher-class reviewers are always treated as having more expertise. Two experimental studies (N = 354 and N = 638) demonstrate that reviewer class plays a causal role in both a contagion process and in an assumption of higher-class knowledge process, but do not provide evidence for the reviewer-participant social matching hypothesis.

## Introduction

Online review platforms play an important role in how people make purchasing decisions, with 82% of U.S. adults indicating that they read online reviews before buying something new [1]. In contrast to traditional “word of mouth” methods for spreading information about an organization, the public, accessible nature of online review platforms allows potential customers to get opinions from a much wider range of people than they would typically encounter face to face. For example, in off-line interactions people typically interact with those who are similar in social class [2] but when using online review platforms people may access opinions from those outside their social circle.

As online review platforms increase potential customers’ access to opinions from a wide range of other people, new questions arise about how people will respond to others from diverse backgrounds in this setting. Research in the fields of communications and social psychology demonstrates two main channels of influence: the “central route,” in which persuasion results from careful and thoughtful consideration of the information presented, and the “peripheral route,” in which persuasion results from simple inferences often based on cues unrelated to the information itself (e.g., whether a review was written by an attractive person)[3, 4]. In this paper, we focus on the role of peripheral cues, specifically on the role of the social class of the reviewers. In face-to-face interactions, people respond differently to others based on their social category membership (e.g., their race, sex, or social class), and similar intergroup processes have been observed in online interactions as well [5]. For example, research has shown that racial bias affects sale prices on online re-sale bidding sites, with African American sellers receiving lower payments than White sellers [6, 7]. In the current research, we ask how people respond to online reviews based on the reviewers’ social class background. As far as we know, this is the first paper to examine such a question in the online review domain.

Study 1 uses observational data from an online review website, Yelp.com. This work explores how the presence and opinions of reviewers from different class backgrounds affect subsequent ratings for a focal organization. We examine how previous reviewers’ class and their opinions about a focal organization shape a focal reviewer’s rating of the organization, and how this differs depending on the reviewer’s own class and the organization’s status. Because this analysis of data from the field cannot determine the causal mechanisms underlying the observed relationships, we then examine responses to reviewer social class in online experiments.

In Studies 2 and 3, we use randomized experiments to test how people respond to reviewers from different social class backgrounds. Participants in the experimental study are presented with one person’s opinion about an organization and asked to make hypothetical judgments about the organization. In this experimental paradigm, we manipulated the reviewer’s apparent social class to be either high or low. This paradigm allows us to establish causal links between the reviewer’s social class and viewers’ responses. We think of these observational and experimental studies as complementary: the observational data demonstrates that these effects exist in a large-scale field data of 1,234,665 reviews submitted by 99,239 reviewers, and the experimental studies provide insights about the causal mechanisms, albeit in a simplified setup.

## Background

Not all reviewers’ opinions are equally influential, and previous research has identified multiple factors that lead people to put more weight into a specific reviewer’s opinion. For example, the presence of individualizing reviewer characteristics (e.g., giving the reviewer’s name and location versus not providing this information) makes a review more influential [8, 9]. Similarly, indicators of a reviewer’s high quality, such as having written a high number of reviews or having received positive feedback from other users (e.g., receiving a “useful” or “helpful” vote), make that reviewer’s opinions more influential [10]. While these studies indicate that potential customers are sensitive to reviewer-level characteristics, they have not examined the role of reviewers’ social group memberships in general or their social class status more specifically.

While people often do not explicitly broadcast their social class, there are typically subtle cues that signal class status. Online review platforms often encourage their users to upload photographs representing themselves, and the information conveyed by these photos allows others to detect the reviewer’s class background [11–14]. Detection of social class status can occur rapidly and automatically, even when people are not aware of it [15–17]. Not only do people effortlessly detect others’ social class, but they also evaluate these groups differently and respond differently to people from higher- versus lower-class groups [18]. Experimental studies allow researchers to isolate the effects of social class by examining how people respond to identical behaviors from someone with higher versus lower social class. In domains such as criminal justice, education, and housing people typically favor those with relatively higher social class status [19–24]. Even in mundane situations, people are more likely to follow the influence of others with relatively higher social class, such as jay-walking more often after a higher- versus lower-class person does so, and offering more help to a woman with a higher-status vehicle than a woman with a lower-status vehicle [25, 26]. Building from this research showing that people with higher social class are generally favored over others with lower social class, here we ask how the class of reviewers affects how potential customers respond to their opinions about an organization.

We examine several possible mechanisms that may drive responses to online reviews. While each mechanism is a separate process, they are not mutually exclusive and may co-occur.

### Influences of reviewer class

One way in which reviewer social class may affect organizational evaluations is through a “contagion” process where the feelings associated with one entity (in this case, the reviewer) then spread to another entity (in this case, the organization) [27, 28]. Previous research has shown that feelings associated with a member of a social group can spread to others that are paired with that person; for example, when an overweight person (a member of a stigmatized social group) is sitting next to an otherwise neutral job applicant in a waiting room, the job applicant is evaluated worse [29]. This phenomenon has been observed with members of a variety of social groups [30, 31] (e.g., race, sexuality, disability), including people displaying stigmatized lower-class behaviors (i.e., using a coupon to save a small amount of money compared to not using any coupons [32]). When applied to the domain of online reviews, we predict that evaluations of a reviewer with high versus low class status may “spread” to evaluations of the organization; if people view the higher-class reviewer more positively than the lower-class reviewer, and the positively-valued higher-class reviewer is paired with the organization, this liking of the reviewer will lead to more positive evaluations of the organization. In this case, we predict that the “contagion” effect would occur if the reviewer themselves was liked, and would occur regardless of the content of the review (i.e., whether the reviewer actually liked the organization or not).

*Hypothesis 1. (“Contagion” hypothesis) Organizations reviewed by higher-class individuals will be evaluated by more positively by third-parties than organizations reviewed by lower-class individuals*.

We also examine an alternative mechanism underlying the influence of reviewers’ class characteristics and test whether higher-class reviewers are more influential because they are perceived as more knowledgeable. Decades of research from social psychology shows that people often assume that someone is an expert based solely on their appearance and their social characteristics [33]. People generally attribute higher competency to people from higher-status social groups, such as the upper class, even when there is no logical reason to do so. Additionally, people who are higher in social class are often “omnivores” who consume from a variety of different types of food, music, etc., rather than focusing on only one favored type [34, 35]. Thus, perceptions that higher class consumers are more knowledgeable consumers may be accurate due to their broad consumption experiences.

*Hypothesis 2. (“Higher class expertise” hypothesis) There will be an interaction between reviewer class and reviewer opinion; a higher-class reviewer’s opinion will be more influential than a lower-class reviewer, and whether he or she endorses or disapproves of the organization will shift the potential customers’ evaluations up or down*.

### Interaction between reviewer class and organization status

Although people typically attribute higher levels of knowledge to people from higher-status social groups, sometimes people from relatively lower-status groups are seen as experts in stereotype-consistent domains; for example, men are treated as “default” experts in many areas, whereas women are treated as experts specifically in female-stereotyped areas such as sewing and childcare [36].

In the online review context, potential customers may be more influenced by a reviewer’s opinion when there is a “match” between the reviewers’ class characteristics and the organizations’ class status. Higher-class reviewers may be seen as experts when evaluating expensive organizations, but lower-class reviewers may be seen as experts in evaluating budget organizations.

One field experiment found that a “match” between an eBay seller’s race (White or Black) and the race-based niche of the products being sold (e.g., a White version of a doll and a Black version of the same doll) led to higher winning prices than when there was a mis-match between the race of the seller and race associated with the product [37]. While this study did not investigate the mechanisms driving the “matching” effect, in our context we predict that reviewers from different class backgrounds will be seen as experts in different areas, their opinions will be more influential in those “matching-class” areas.

Hypothesis 3. (“Organization-reviewer status matching” hypothesis) There will be an interaction between organizations status, reviewer class and reviewer opinion; for expensive organizations, a higher-class reviewer’s opinion will be more influential than a lower-class reviewer, and whether he or she endorses or disapproves of the organization will shift the potential customers’ evaluations up or down; and vice versa for budget organizations.

While Hypotheses 2 and 3 make identical predictions for when an organization is higher class, they diverge in their predictions about whose opinion will be more influential for a lower-class organization.

### Interactions between reviewer class and viewer’s class

People may also respond differently to a reviewer’s social class depending on the individual’s own social class. A large body of work from social psychology demonstrates that people like others with whom they share a group membership [38, 39]. Looking at social class specifically, people affiliate more with others who share their social class background, particularly when they are higher- or lower-class [40]. Thus, we consider whether people who are reading online reviews are most strongly influenced by reviewers who share their social class background.

Hypothesis 4. (“Existing reviewer- focal viewer status matching” hypothesis) There will be an interaction between participant class, reviewer class, and reviewer opinion; for a lower-class participant, a lower-class reviewer’s opinion will be more influential than a higher-class reviewer; vice versa for a higher-class participant.

## Study 1

### Setting and data source

In Study 1, we test our hypotheses on large-scale observational field data. We utilized the publicly available dataset of online reviews provided by Yelp.com as part of the 8^th^ Yelp challenge. The Yelp challenge is a bi-yearly competition organized by Yelp.com, an online review website. In the challenge, Yelp.com shares some of their review data with the public and ask researchers “to conduct research or analysis on our data and share their discoveries with us.” (see https://www.yelp.com/dataset/challenge, accessed on 9/3/2018). The competition is primary aimed at college students, and the company awards $5000 to each of the ten top submissions by students; we used this dataset after the competition was completed because the public dataset allowed us to access accurate, detailed information that would otherwise be unattainable.

Yelp.com accepts reviews for all kinds of organizations, from shopping centers to seafood restaurants, or from Mexican restaurants to music venues, but roughly 85% of all reviews are about food and dining establishments. The data contains, for each review, the date of the review, the user ID of the reviewer, the ID of the organization being reviewed, a star rating ranging from 1 star to 5 stars, and the full text of the review (if submitted). We also have information available about the name of the organization reviewed, its location, its type and categorization (e.g., Mexican restaurant), and its price range. The price information is provided in four categories, denoted with dollar signs ($—‘cheap’, 26% of the sample, $ $ ‘moderate’, 61% of the sample, $ $ $ ‘expensive’, 10.5%, or $ $ $ $ ‘splurge’, 2.5%). (When posting a review, Yelp.com provides reviewers with the option to “Have additional tips on this organization,” such as the price range or whether the organization accepts credit cards or how expensive it is. When an organization receives enough votes about the price range and there is enough agreement on the votes, Yelp displays the price.) The Yelp.com data have been analyzed before in the literature, see e.g. [41–45].

### Sampling strategy

For the purposes of our analyses, we restrict our sample along three dimensions. First, in order to have enough observations per organization and per reviewer to establish their social class, we restrict our sample to reviewers and organizations with at least five reviews. Second, because price is crucial in our analyses, we exclude from our sample organizations for which we have no price information. Third, we restrict our sample to the U.S. locations (Pittsburgh, Charlotte, Urbana-Champaign, Phoenix, Las Vegas, Madison, Cleveland). See Table 1 for descriptive statistics (some of the variables shown in this table will be explained below).

**Table 1:** Descriptive statistics of variables used in Study 1 (based on the sample for model 4).

Variable	Mean	Std. Dev.	Min	Max
Rating	3.811	1.213	1	5
Review year	2013.645	1.868	2007	2016
Price ($ = 1, $ $ = 2, $ $ $ = 3 $ $ $ $ = 4)	1.885	0.631	1	4
Focal Reviewer’s Class	1.871	0.393	1	4
Prior mean ratings by lower-status Reviewers	3.830	0.510	1	5
Prior mean ratings by higher-status reviewers	3.837	0.534	1	5

N = 941,678

### Measures and methods

#### Variables

Dependent variable: Star rating. The primary dependent variable is the star rating a focal reviewer gives to an organization.

Independent variable: Organizational status. As price level is a generally used cue for the status or prestige of a brand or an organization [46], we proxy the status of the organizations with their assigned price level. As data are only available about the price levels at the date when the dataset was created, the status levels of the organizations are assumed to be constant.

**Fig 1:**
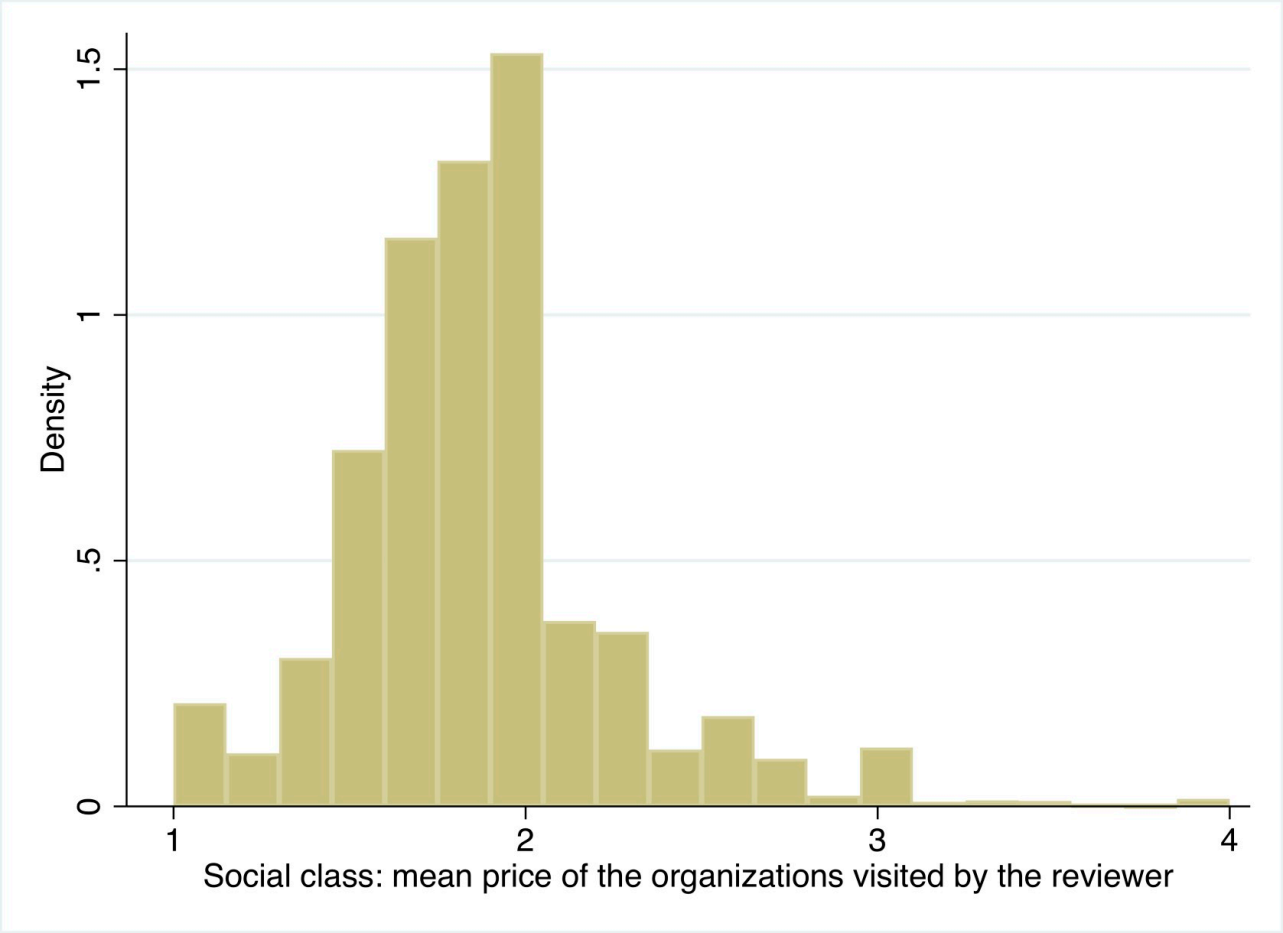
Study 1: Distribution of social class: Mean price of the organizations visited by the reviewer.

Independent variable: Social class of the reviewers. We proxy the social class of reviewers by the average price level of the organizations they review. For example, if a reviewer had visited a 10 “one dollar sign” restaurants and 2 “two dollar sign” restaurants, their continuous class measure will be (10*1+2*2)/12 = 1.16. This approach rests on a long stream of consumption research in sociology and marketing, documenting that higher social class typically correlates with higher level of consumption of more expensive and luxury items [47, 48]. Fig 1 shows the distribution of the reviewers’ class as determined by the mean price of organizations they review.

In certain analyses below, we will use a dichotomous version of the social class variable. Specifically, we will split the reviewers by the median of the distribution, and call the reviewers lower/higher class if they were below/above the median split.

We note that the analyses of Study 1 assume that the focal reviewer is somewhat aware of the social class of other reviewers. We can see multiple ways in which this could happen: (1) by inferring the social class of the reviewer from their profiles, (2) by inferring the social class of the reviewer from their profiles, and/or (3) by inferring the social class of the reviewer from the kind of organizations and restaurants they review. First, the focal reviewer can use the name, profile photo of other reviewers to infer their social class. For example, a profile photo with expensive clothing/sunglasses/car/house may signal social class [11, 12]. Reviewer profile photos are available on most review websites. [Note that in Studies 2 and 3 we demonstrate experimentally that the same reviewer, holding everything else constant, is judged as higher class if his profile picture contains an expensive-looking versus a more modest-looking house.] We acknowledge that these names and profile photos are typically self-reported and therefore are not necessarily true–but what matters to us is not whether they are true, rather, whether future reviewers use them to infer social class. Second, people may use the content and style of the reviews to infer the social class of reviewers. For example, a person using proper grammar will be viewed as more educated and higher class than someone whose text include multiple spelling mistakes. Third, people may infer the social class of others from the kind of organizations and restaurants they review. Specifically, we think that someone who typically reviews expensive and high-class restaurants will be seen as higher class while someone who typically reviews low-class restaurants such as chain diners and hot dog restaurants, will be seen as lower-class [49]. We note that we do not measure (1) and (2). Our measures purely rest on (3), and we make the assumption that these three types of signals would point to the same direction. We acknowledge that these assertions should be tested in future research.

Additional control variables. To control for alternative explanations and heterogeneities across locations and organization types, in all the models we include fixed effects for organization type (the categories used by Yelp; e.g., Restaurants, Shopping, Spas, Automotive, etc.), location, and year of review. To control for further quality differences across organizations, we include in the models the mean of all the star ratings the organization received prior to the focal review (note that this approach is better in capturing organization quality than an organization fixed effect because it allows for over time variations, such as a restaurant becoming higher quality over time).

#### Estimation methods

We estimate OLS models, in which the dependent variable is the star rating the focal reviewer gave to the organization, and the independent variables are based the organization’s previous reviews, as calculated from prior to the focal review. For example, for a review written about the restaurant “Arrogant Butcher” on February 3^rd^, 2014, we assess the restaurant’s characteristics by looking at only the reviewers who posted reviews about this restaurant prior to February 3^rd^, 2014.

### Results

Using OLS, we test if reviewers give higher ratings to an organization if it is frequented by (in our context: reviewed by) other reviewers that are relatively higher versus lower class (H1); whether reviewers are more influenced by prior reviews of higher class reviewers (versus prior reviews of lower class reviewers) (H2); and whether these effects are moderated by the organization’ status (H3) or by the focal reviewers’ own class background (H4).

Table 2 shows the results. Model 1 shows that, on average, having a higher-class clientele increases the ratings the organization gets. This finding is robust across later specifications in Models 2 to 4. These findings are consistent with Hypothesis 1 (the “Contagion” hypothesis) in that the mere presence of higher-class reviewers predicts higher rating by the focal reviewer.

**Table 2:** Study 1: OLS regressions. Dependent variable: focal star rating. Independent variables: the reviewed organization’s price level, the social class composition of the existing clientele, the mean of prior star ratings, and the focal reviewer’s social class.

	(1)	(2)	(3)	(4)
Price	0.016***	0.010***	0.117***	0.011***
	(0.002)	(0.003)	(0.017)	(0.003)
Prior Reviewers’ mean class	0.063***	0.110***	0.108***	0.114***
	(0.006)	(0.009)	(0.009)	(0.009)
Prior mean ratings by all	0.748***			
	(0.002)			
Prior mean ratings by lower-status reviewers		0.409***	0.641***	0.720***
		(0.004)	(0.012)	(0.018)
Prior mean ratings by higher-status reviewers		0.418***	0.240***	0.107***
		(0.004)	(0.011)	(0.018)
Price X Prior mean ratings by lower status reviewers			-0.126***	
			(0.006)	
Price X Prior mean ratings by higher status reviewers			0.099***	
			(0.006)	
Focal reviewer class				-0.020***
				(0.024)
Focal reviewer class				-0.169***
X Prior mean ratings by lower status reviewers				(0.010)
Focal reviewer class				0.171***
X Prior mean ratings by higher status reviewers				(0.010)
Constant	0.835***	0.323***	0.103	0.331***
	(0.289)	(0.062)	(0.069)	(0.076)
Observations	1,234,665	941,678	941,678	941,678
R-squared	0.127	0.120	0.121	0.121

Bootstrapped standard errors in parentheses

*** p<0.01

** p<0.05

* p<0.1

All models include organization category, year, and city fixed effects.

While the previous model included the prior rating of the organization by all reviewers, Models 2 to 4 examine the influence of previous ratings by “low” and “high” class reviewers separately. We split the overall “prior rating” for the organization variable into two variables: “Prior mean ratings by lower class reviewers” and “Prior mean ratings by higher class reviewers” (as discussed in the “Measures and Methods” section for Study 1, we obtain the lower/higher class reviewers by splitting the reviewers at the median of the distribution of social class variable). To explore effects of both types of reviewers simultaneously, as a pre-specified exclusion criterion, we exclude on observations where the organization has received less than five reviews from both lower-class and higher-class reviewers (therefore the sample size drops from 1,234,665 to 941,678 in Models 2 to 4).

In Models 2 and 3 we test Hypotheses 2 and 3. Hypothesis 2, the (“Higher class expertise” hypothesis, states that a higher-class reviewer’s opinion will be more influential than a lower-class reviewer’s opinion. Hypothesis 3, the “Organization-reviewer status matching” hypothesis, specifies hypothesis 2 and predicts that the opinion of higher-status reviewers will only matter more for more expensive organizations, with the opposite pattern seen for lower priced organizations.

Model 2 tests Hypothesis 2. The results show that opinions from both groups influence a focal reviewer’s rating (the estimate for “Prior mean ratings by lower-status reviewers” is b = 0.409, SE = 0.004; while the estimate for “Prior mean ratings by higher-status reviewers” is b = 0.418, SE = 0.004, both significantly different from 0 at p<0.01). To test whether these two coefficient estimates differ from each other significantly, we conducted an F-test that restricts the two coefficients to be the same. This test revealed that the two coefficients are not significantly different (p = 0.187). This finding disproves Hypothesis 2, i.e., contradicts the overall prediction that the opinion of higher-status reviewers will be on average more influential.

**Fig 2:**
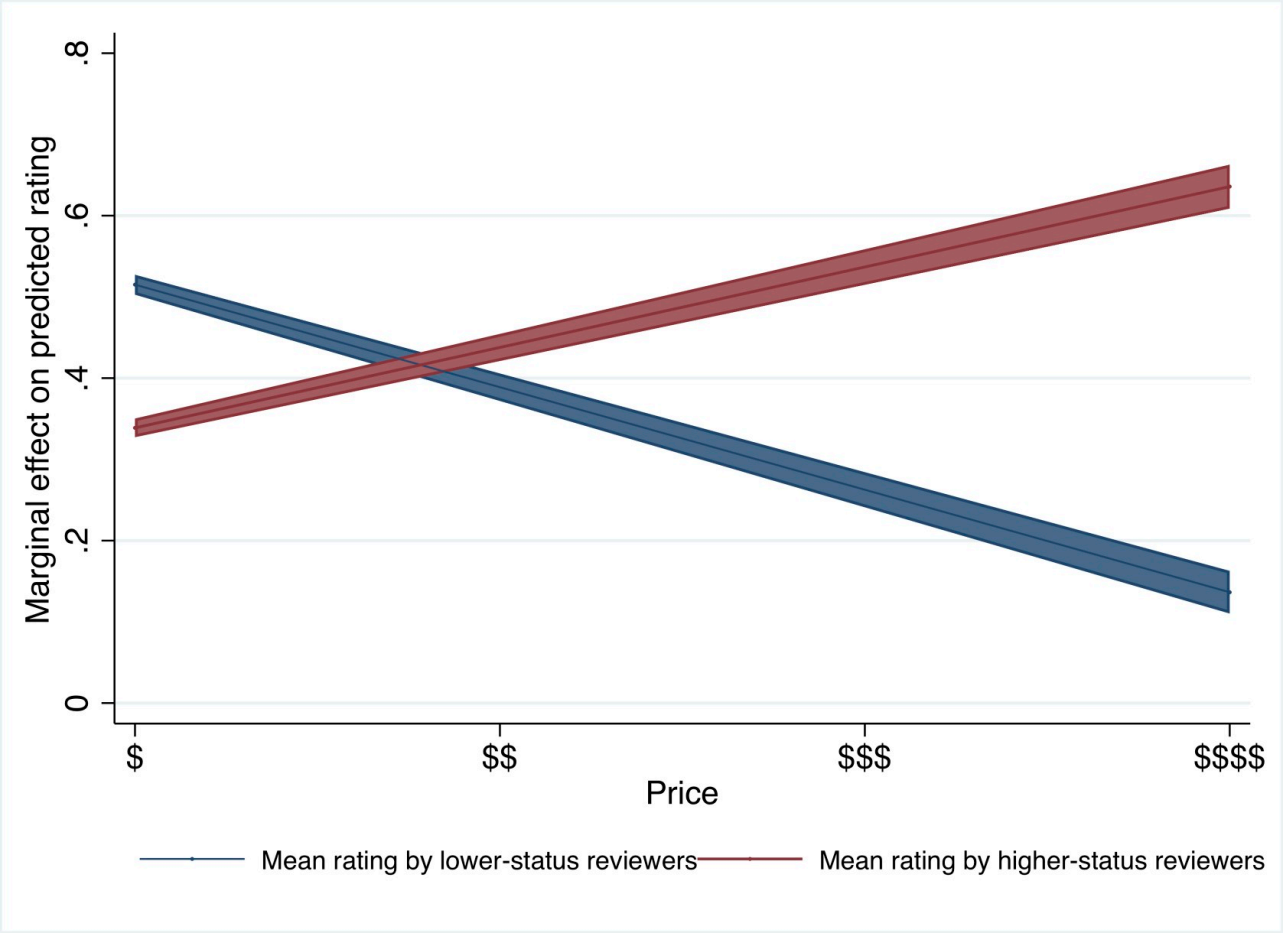
Marginal increase on a focal reviewer’s rating predicted by prior ratings by higher- and lower-class reviewers as a function of the organization’s price level. **Study 1:** Based on Model 3 in Table 2. The lines represent linear regression estimates, the shaded areas show the 95% confidence intervals.

Model 3 sets out to test Hypothesis 3, the “Organization-reviewer status matching” hypothesis, and tests whether for expensive organizations, a higher-class reviewer’s opinion will be more influential than a lower-class reviewer’s opinion, and whether he or she endorses or disapproves of the organization will shift the potential customers’ evaluations up or down; and vice versa for budget organizations. To test this hypothesis, Model 3 includes the interactions between the price level of the organization and prior ratings by lower and higher-class reviewers. The positive and significant interaction effect between “Price” and “Prior mean Rating by Higher status reviewers” indicates that the higher the price level of the organization, the more emphasis reviewers put on ratings by higher-class reviewers and the less emphasis they put on ratings by lower-class reviewers. Similarly, for lower priced organizations, lower-class reviewers’ ratings are more influential than higher-class reviewers’ ratings. In other words, we observe a matching phenomenon where the reviewers who match the organizations’ class are most influential. (We note that the coefficient estimates are significantly different from each other at p<0.001 according to an F-test both between the main effects and also between the interaction effects). Fig 2 visualizes the results. These findings confirm Hypothesis 3. These findings also explain why we did not find significant overall effect of reviewers’ social class: the opinion of higher-class reviewers is more influential than the opinion of lower-class reviewers when expensive organizations are reviewed ($ $ $ or $ $ $ $ price level), but less influential when low-price organizations are reviewed ($ price level)–these effects cancel each other out when averaged for the whole population.

**Fig 3:**
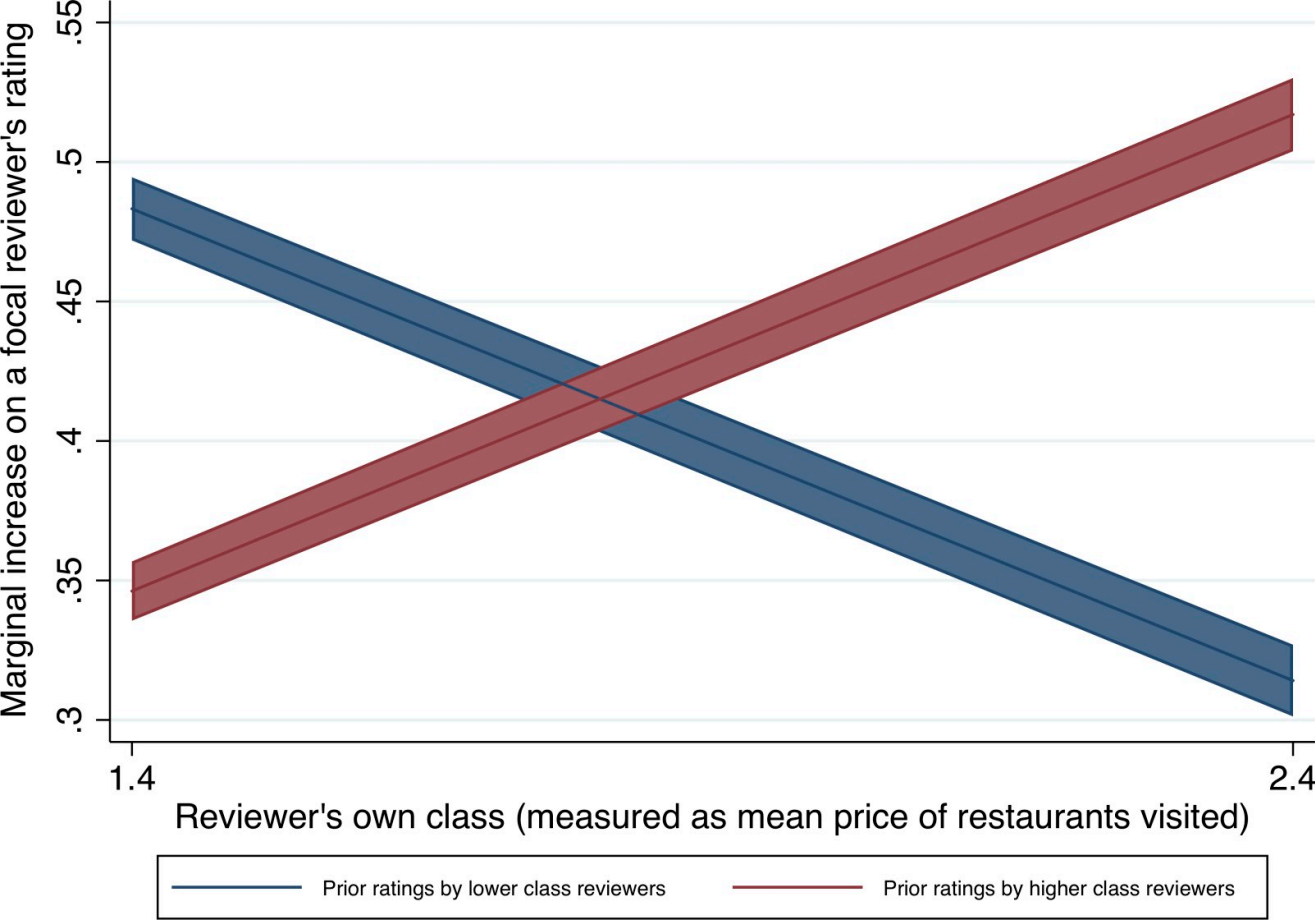
Study 1: Marginal increase on a focal reviewer’s rating predicted by prior ratings by higher- and lower-class reviewers as a function of the focal reviewer’s own class. Based on Model 4 in Table 2. The lines represent linear regression estimates, the shaded areas show the 95% confidence intervals.

In Model 4, we investigate the role of the focal reviewers’ own social class as a predictor of how they will respond to other reviewers from different class backgrounds. This allows us to test Hypothesis 4 (the “Reviewer-participant status matching” hypothesis), which predicted that reviewers would be most influenced by the opinions of those who share their social class background. Specifically, in Model 4 we interacted the reviewer’s own class with prior ratings by higher- and lower-class reviewers. We found that the higher the reviewer’s own class background, the more their opinions matched those of higher-class reviewers and the less they matched those of lower-class reviewers. (We note that the coefficient estimates are significantly different from each other at p<0.001 according to an F-test both between the main effects and between the interaction effects). See Fig 3 for a graph of how high- and low-class reviewers’ influence differs depending on the focal reviewer’s class. These results confirm Hypothesis 4.

#### Discussion

An analysis of field data from a large online review dataset demonstrated strong patterns consistent with three of our hypotheses. First, in support of a “contagion” hypothesis, organizations with relatively more higher-class reviewers were given more positive ratings, controlling for overall price and prior ratings. Second, in support of a “matching” hypothesis, ratings by higher-class reviewers were more influential than ratings by lower-class reviewers for more expensive organizations, but not for cheaper organizations. Third, in support of a “same-class” hypothesis, reviewers who were higher in class were more influenced by previous higher-class reviewers while reviewers who were lower in class were more influenced by previous lower-class reviewers.

These findings show that not all reviewers’ opinions are equally relevant, and that examining reviewers’ social class can allow for unique predictions about responses to an organization. Our conclusions are limited by the observational nature of our field study. For example, perhaps the increased influence of higher-class reviewers is not due to their class background but instead comes from a more persuasive writing style in their written reviews? Perhaps the higher-class reviewers in our sample also differ from lower-class reviewers on race, adding a potential confound? As a more direct test of the influence of higher-class reviewers, we next conduct experimental tests of higher- and lower-class reviewers’ influence in a controlled setting, allowing us to eliminate the possibility that our results were due to confounding variables.

## Study 2

This experiment tests whether visitors to online review platforms are sensitive to reviewer’s social class and examines the ways in which reviewer class characteristics shape people’s perceptions an organization’s quality. In our experimental paradigm, we present a sample of online participants with a fictional online review page for an organization. The fictional page includes a quote from a featured reviewer; within this design, we manipulate the reviewer’s apparent social class and the organization’s status as a luxury or budget organization. In this study, we examine responses to a review of an automotive dealership because automotive brands are often associated with social class status.

The benefit of our experimental design is that we could keep consistent across conditions many aspects of the organization, the content of the review, and the reviewers’ identity (e.g., name, appearance). After participants viewed the fictional review page, we assessed participants’ opinions about the organization and the reviewer.

### Method

#### Participants

To determine the desired sample size, we conducted a power analysis for ANOVA with the G*Power Software. We conducted the power analyses based on the ANOVA design with the experimentally manipulated variables (Study 2: 2X2 and Study 3: 2X2X2). We ran our calculations using what is considered to be a “medium” effect size (.25), a conservative 95% power (beta) and 5% error rate (alpha). The power analysis suggested that we collect a minimal N = 200, which we doubled so that we can reliably estimate mediation models as well. We recruited 400 U.S.-based online participants from Amazon’s Mechanical Turk platform. Out of the 400 participants who completed the experiment, 46 did not pass a pre-determined attention check (as described below), and therefore we excluded them from further analyses. We did not find evidence that the exclusion rate would differ across conditions. Our final sample was 354 participants: 60% male and 40% female, predominantly White (72%), self-identified as middle class (lower class, 19%, lower-middle class, 31%, middle class, 42%, higher-middle class, 7%, higher class, 0.3%), and in middle adulthood (*M* = 35.83 years, *SD* = 10.58 years, range = 18–74). All demographic identification is self-reported by participants. Yale University’s IRB Board approved this study (approval # 1508016387). Each participant completed an online consent form and clicked to indicate their consent before beginning the study.

### Procedure

Participants read a brief description of the study procedure and were randomly assigned to one of four conditions in the 2 (Organization Status: High, Low) x 2 (Reviewer Class: Higher, Lower) between-subjects design. The participants were randomized into one of the four conditions by the Qualtrics software (100 in each condition). Participants saw one fictional review webpage for an automotive organization that gave some basic information about the store and included a quote about the store from a “featured reviewer.” The stimuli used in this experiment are available from the corresponding author at request.

The High or Low status of the automotive organization was conveyed via the automotive brand, which was featured in the organization name (Apex *BMW* [*Kia*] Sales and Service), and in the reviewer’s quote. The Higher or Lower class status of the reviewer was conveyed via photograph and via the type of car owned (i.e., Mercedes, Toyota). The reviewer’s photograph was constructed using photo editing; it consisted of a man’s face, which was the same in both conditions, in front of a house that was either small and run down, conveying lower class, or in front of a house that large and well-kept, conveying higher class.

The reviewer’s quote contained information about both organizational and reviewer status information. The quote read, “I was driving nearby and my engine started smoking. Even though I don’t drive a *BMW* [*Kia*], the guys here were great and took a look at my *new Mercedes* [*old Toyota*]. The mechanics really know their stuff! But in the end I needed to be towed to another garage.”

To ensure that participants were paying attention to the study, after fifteen seconds of viewing the store information participants were asked to recall the name of the organization in an open-ended response; those who could not recall the name were excluded from the sample due to inattentiveness.

While viewing the review page, participants responded to five statements about the automotive store’s quality, using a 0–100 scale, presented in a random order (“I think this organization would provide high quality service,” “…timely service,” “…do a good job,” “I would trust the people at this organization,” and “I think I would get bad service at this organization” reverse scored). Responses to these five statements were closely related (alpha = .92) and thus were averaged to create a liking rating for the organization. Next, we asked participants to evaluate the reviewer about his knowledge with cars. To ensure our reviewer class manipulation was interpreted in line with our intentions, as a manipulation check, we asked participants “In terms of social class, how would you describe the reviewer?” For this question, we did not provide any definition of social class, and participants answered the question by moving a slider scale from 0 (Lower Class) to 100 (Higher Class). Last, participants answered demographics questions.

### Results

Responses to the manipulation check, the organizational evaluation questions, and the reviewer knowledge question were analyzed using ANOVAs with Reviewer Class and Organization Status as independent variables. On the manipulation check, as intended, participants rated the Lower Class reviewer as lower in class (M = 34.93, SD = 18.91) than the Higher Class reviewer (M = 72.52, SD = 16.88), *F*(1, 350) = 394.79, *p* < .001. Unexpectedly, the reviewer was rated as lower in class when the organization was High status (M = 51.33, SD = 25.98) compared to Low status (M = 55.69, SD = 25.98), *F*(1, 350) = 5.78, *p* = .017.

Turning to our hypothesis, we find that the Organizational Evaluation scores revealed that participants were more positive about the organization when the reviewer was Higher class (M = 70.34, SD = 15.84) relative to Lower class (M = 64.50, SD = 19.05), *F*(1, 350) = 9.84, *p* = .002, partial η^2^ = 0.03. This finding confirms Hypothesis 1 (the “contagion” hypothesis).

We did not observe an effect of organizational status or an interaction effect, both *p*s > .46. This finding disconfirms Hypothesis 3, the “Organization-reviewer status matching” hypothesis.

**Fig 4:**
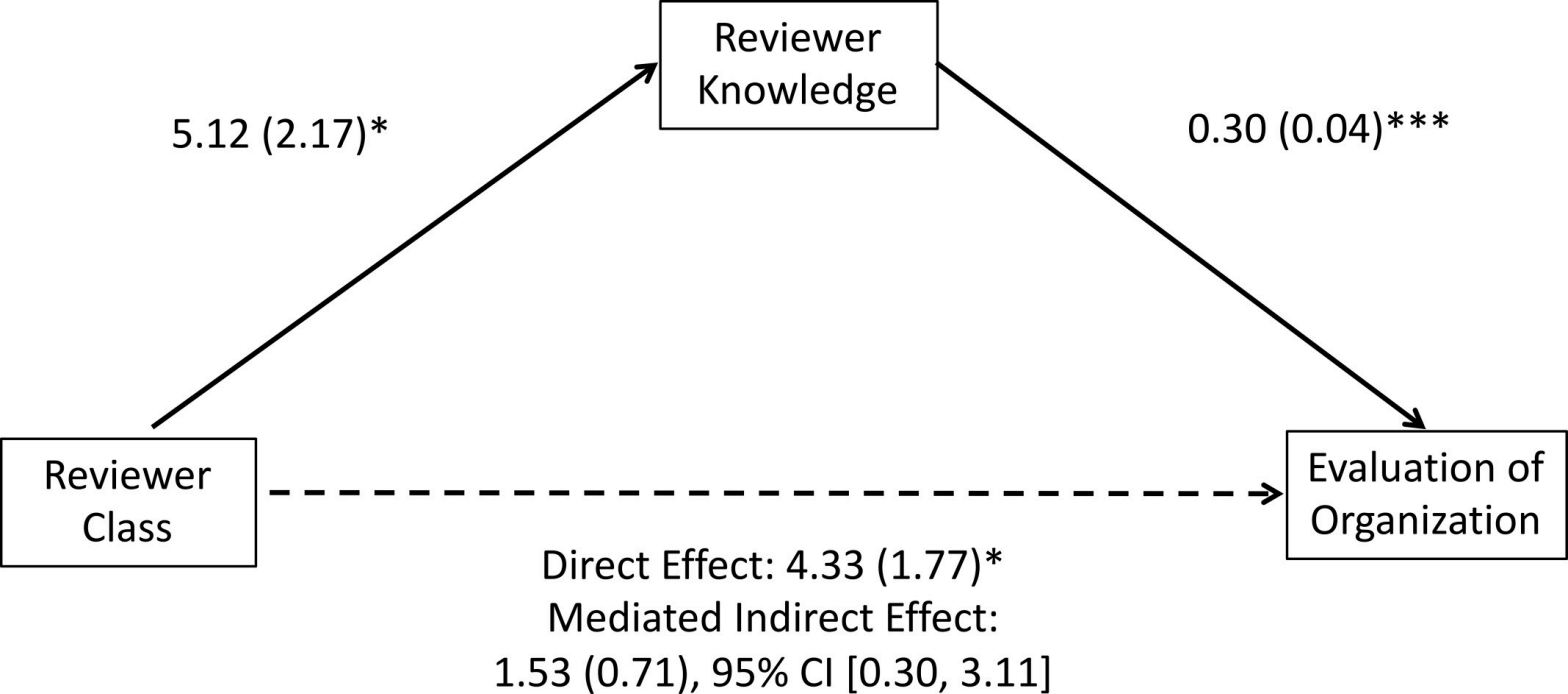
Coefficients and standard errors in a bootstrapping mediation analysis from Study 2, with Organization Status included as a covariate. * indicates *p* < .05, *** indicates *p* < .001.

We next examined the reviewer knowledge rating and found that participants thought the Higher Class reviewer had more knowledge (M = 43.58, SD = 21.40) than the Lower Class reviewer (M = 38.44, SD = 19.32), *F*(1, 350) = 5.58, *p* = .019, partial η^2^ = 0.02, with no effect of organizational status or an interaction effect, both *p*s > .28. We further examined whether the effect of Reviewer Class on Organizational Evaluations was mediated by reviewer knowledge, using a bootstrapping mediation (PROCESS macro for SPSS [50], model 4); because Organizational Status was not a significant predictor of evaluations in the ANOVA analyses, it was entered into the bootstrapping mediation as a covariate. The results, shown in Fig 4, revealed the predicted indirect effect, indicating that changes in perceived reviewer knowledge partially explain changes in evaluations of the organization. Although perceived reviewer knowledge mediated the relationship between reviewer class and evaluations of the organization, the direct effect remained significant, suggesting that other factors may also explain this relationship. These patterns provide evidence for Hypothesis 2, the “Higher class expertise” hypothesis.

Finally, we examined the role of participants’ own social class in shaping their responses to our experimental conditions. We analyzed Organizational Evaluation scores with an ANOVA that included both the experimental variables and participants’ self-reported social class (categories coded 1–5). While the Reviewer Class effect was still significant in this model, *F*(1, 335) = 8.36, *p* = .004, there was neither a main effect of participant class nor any interaction effects, all *p*s > .21. This pattern disconfirms Hypothesis 4. “Reviewer-participant status matching” hypothesis)

### Discussion

Study 2 experimentally demonstrates that people prefer organizations with reviewers who are relatively higher in social class compared to reviewers with lower social class, regardless of whether the organization is higher or lower in status, and regardless of the participant’s self-reported class. We did not observe an interaction between reviewer class and organization status, nor an effect of participant class on participants’ responses.

These results are consistent with Hypothesis 1, the contagion of positivity process, as observed in Study 1 as well as Hypothesis 2, where higher-class reviewers would be seen as experts in general. While Hypothesis 1 and Hypothesis 2 each suggest a different mechanism, these two processes may occur in tandem. The effect of reviewer class on organization evaluations was partially mediated by perceptions of reviewer knowledge, indicating that reviewer knowledge explains some of the observed effect and is consistent with the idea that both processes may be happening (in Study 3, we provide more detailed evidence for this).The results do not offer support for the idea that reviewers are most influential in class-matched organizations (Hypothesis 3), nor for Hypotheses 4. We discuss the possible reasons for these non-findings in the Discussion section of Study 3.

## Study 3

The next experiment replicates the previous findings in a new domain–namely, eyeglass stores–and extends the scope of the research by exploring two possible mechanisms for the effects observed in Study 2. We test whether higher-class reviewers always increase positive evaluations of the organizations, as predicted by the “contagion” Hypothesis 1, or whether higher-class reviewers wield more influence on organizational evaluations and thus can decrease positivity if they convey negative opinions, as predicted by the “knowledge” Hypothesis 2. If both processes occur in tandem, these two opposing processes may appear to “cancel each other out” when a reviewer expresses a negative opinion, and thus the influence of negative evaluations of an organization would be equivalent across reviewer class.

To this end, we conducted an experiment with a 2 (Reviewer Class: Higher, Lower) x 2 (Review Content: Positive, Negative) x 2 (Organization Status: High, Low) between-subjects design. We presented participants with a fictional review webpage for an eyeglass store that was either Luxury or Budget, and which featured a reviewer who was portrayed as either higher- or lower-class, and who either endorsed the store’s quality or did not. After participants viewed the fictional review page, we assessed participants’ opinions about the store and the reviewer.

Rather than just asking about the reviewer’s knowledge, in this experiment we asked participants to rate the reviewer on three dimensions (liking, knowledge, and trust). We selected these dimensions because we expected that each could play a role in the different hypothesized mechanisms. We expected that general liking would most closely align with the “Positive Contagion” process, as positive feelings toward the reviewer are critical in order for positive feelings to transfer to the organization being reviewed. We expected that the Knowledge and Trust questions would most closely align with a “knowledge” process. We use two questions to probe different aspects of knowledge and capture two possible dimensions–our knowledge question asks specifically about knowledge in the specific retail domain being reviewed, while the trust question elicits more general evaluations about whether the reviewer’s opinions should be adopted.

### Method

#### Participants

To determine the desired sample size, we conducted a power analysis for ANOVA with the G*Power Software. The power analysis suggested that we collect a minimal N = 405, which we doubled so that we can reliably estimate mediation models as well. We recruited 811 U.S.-based online participants from Amazon’s Mechanical Turk platform. Out of the 811 participants who completed the experiment, 173 did not pass a pre-determined attention check (as described below), and therefore we excluded them from further analyses. Our final sample was 638 online participants recruited from Amazon’s Mechanical Turk platform; we excluded those who did not pass the manipulation check. (We did not find evidence that the exclusion rate would differ across conditions). The final sample was 53% male and 46% female with three participants choosing not to answer; predominantly White (80%); self-identified as lower class (17%), lower-middle class (28%), middle class (47%), higher-middle class (7%) and higher class (0.6%), with two participants choosing not to answer; and in middle adulthood (*M* = 36.60 years, *SD* = 11.28 years, range = 18–81) with one participant choosing not to answer. All demographic identification is self-reported by participants. Yale University’s IRB Board approved this study (approval # 1508016387). Each participant completed a consent form and clicked to indicate their consent before beginning the study.

#### Procedure

Participants were presented with a fictional online review webpage for an eyeglass store. This page gave some basic information about the store and included a quote about the store from a “featured reviewer.” Participants were randomly assigned to one of eight conditions in our 2 (Reviewer Class: Higher, Lower) x 2 (Review Content: Positive, Negative) x 2 (Organization Status: High, Low) between-subjects design. The stimuli used in this experiment are available from the corresponding author at request.

The Higher or Lower-class status of the reviewer was conveyed via photograph, as in Study 2, and via occupational information. In the quote, the reviewer disclosed occupational information conveying his lower-class status (cashier) or higher-class status (engineer).

The reviewer either expressed a Positive or Negative view of the organization, as indicated by the number of “stars” given to the organization (2 out of 5 stars vs. 5 out of 5 stars) and by the content of the reviewer’s quote about the organization. In the Positive [Negative] condition the quote read, “I was nearby and my glasses broke. I’m an *engineer/cashier* and I need my glasses everyday, and the staff here *were able to fix my glasses right away* [could not fix my glasses right away]. *They were knowledgeable and friendly* [They were clueless and a bit rude].”

The High or Low status of the eyeglass store was conveyed via the name of the store (Apex *Luxury* [*Budge*t] Optics), a tagline (“The latest fashions *from luxury brands* [*at budget prices*], for your eyes”), a price symbol ($ vs. $ $ $) and with two photographs. After fifteen seconds of viewing the store information participants were given the attention check question asking them to recall the store name.

While viewing the store’s review page, participants responded to several questions. First, participants were presented with four statements, in a random order, about the store’s quality (“I think this organization would provide high quality service,” “…timely service,” “…do a good job,” “I would trust the people at this organization”). Participants indicated their agreement using a scale from 0 (not at all) to 100 (very much). Because responses to these four statements were closely related (alpha = .97), responses were averaged to create a liking rating for the organization.

To ensure our organizational status manipulation was interpreted in line with our intentions, as a manipulation check, participants were asked, “How much do you think a pair of eyeglasses would cost at this organization, on average?”

Next, participants were asked to evaluate the reviewer by responding to three questions, in a random order, using 0–100 scales (“How much do you like the reviewer?” “How much do you trust the reviewer?” “In your opinion, how much does the reviewer know about eyeglasses?”).

To ensure our reviewer class manipulation was interpreted in line with our intentions, as a manipulation check, participants were asked, “In terms of social class, how would you describe the reviewer?” and answered on a scale from 0 (Lower Class) to 100 (Higher Class). As in Study 2, we did not provide any definition of social class. Last, participants answered demographics questions about themselves, including a 0–100 social class slider like the one used to evaluate the reviewer.

### Results

#### Manipulation checks

Responses were analyzed using an ANOVA with Reviewer Status, Review Positivity, and Organization Status as independent variables. Responses to the manipulation check questions revealed that our stimuli were interpreted as intended. Participants thought that eyeglasses would be less expensive at the Lower Status store (M = 92.24, SD = 59.70), compared to the Higher status store (M = 197.90, SD = 127.48), *F*(1, 630) = 188.30, *p* < .001, with no other main effects or interactions.

As intended, participants rated the reviewer’s class lower in the Lower-class reviewer conditions (M = 32.38, SD = 18.49) than the Higher-Class reviewer conditions (M = 68.66, SD = 16.25), *F*(1, 630) = 696.06, *p* < .001. Unexpectedly, there was also a main effect of review positivity on reviewer class, such that reviewers who gave Positive reviews were seen as higher in class (M = 52.65, SD = 24.98) than those who gave Negative reviews (M = 48.31, SD = 25.16), *F*(1, 630) = 7.72, *p* = .006. This result also raises the possibility that, in addition to occupational and material wealth cues to social class, people’s public opinions may also shape how their class is perceived.

#### D*ependent variable 1*: *Evaluations of the organization*

First, we examined Organization Evaluations. We observed the predicted main effects., The organization was evaluated more positively in the Positive review condition relative to the Negative review condition, *F*(1, 630) = 643.52, *p* < .001, partial η^2^ = 0.51, showing that people’s evaluations are sensitive to information from online reviewers. The organization was evaluated more positively in the High Organization Status condition relative to the Low Organization Status condition, *F*(1, 630) = 8.20, *p* = .004, partial η^2^ = 0.01, showing that people value status in this domain.

We also find that the reviewer’s status has an overall positive effect on evaluation, such that the Organizational Evaluation is higher in the Higher-Class Reviewer conditions than in the Lower-Class Reviewer conditions (F(1, 630) = 5.51, *p* = .019).

**Fig 5:**
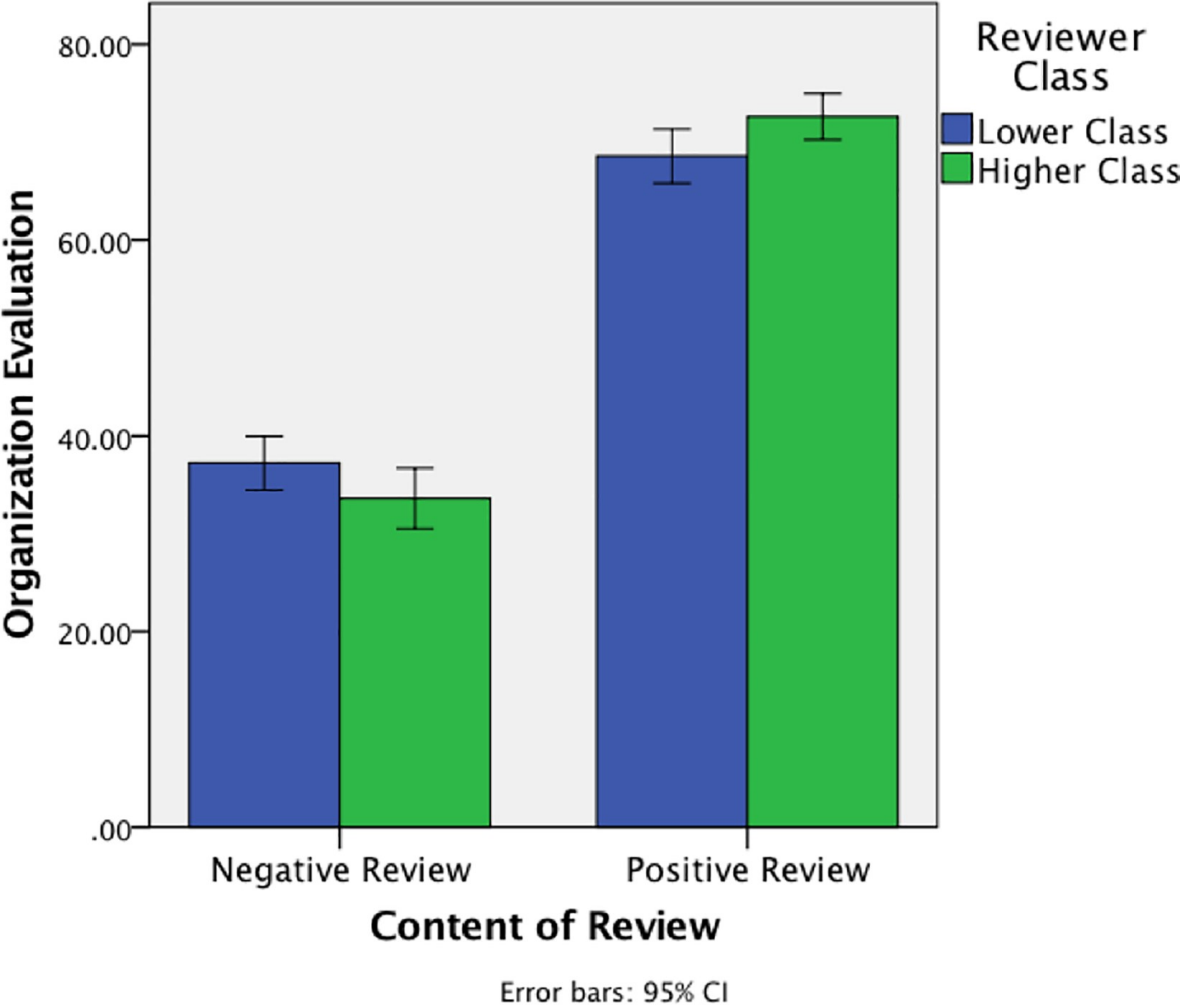
Means from Study 3 showing the interaction between review Positivity and Reviewer Class, collapsed across Organization Status conditions.

Next we discuss two-way interactions. There was an interaction between review Positivity and Reviewer Class, *F*(1, 630) = 7.61, *p* = .006, partial η^2^ = 0.01, shown in Fig 5. We further analyzed this interaction by examining the Positive and Negative reviews conditions separately.

In the Positive review conditions, participants viewed the organization more positively when the reviewer was Higher Class (M = 72.62, SD = 15.26) relative to Lower class (M = 68.56, SD = 17.80), *F*(1, 321) = 4.73, *p* = .030, partial η^2^ = 0.02; this is consistent with Hypothesis 1, that positivity associated with higher-class reviewers “spreads" to associated organizations. When the organization had Higher status (M = 73.04, SD = 15.11) relative to the Lower status (M = 68.43, SD = 17.71), *F*(1, 321) = 6.40, *p* = .012, partial η^2^ = 0.02, consistent with past work showing that people evaluate luxury brands positively, but there was no interaction between the two variables. In contrast, in the Negative review condition, there was not a significant main effect of reviewer class (Higher class, M = 33.60, SD = 19.50; Lower class, M = 37.22, SD = 17.64), *F*(1, 309) = 3.08, *p* = .08. We offer a more in-depth interpretation of this non-significant result, and it fits with our hypotheses, in the Discussion section.

#### Dependent variable 2: Evaluations of the reviewer

We next examine how participants evaluated the reviewer in each condition. Participants liked the Higher Class reviewer (M = 56.51, SD = 19.24) more than the Lower Class reviewer (M = 52.61, SD = 21.70), *F*(1, 630) = 5.18, *p* = .023, partial η^2^ = 0.01, liked the Positive reviewer (M = 59.58, SD = 22.11) more than the Negative reviewer (M = 49.34, SD = 19.01), *F*(1, 630) = 38.92, *p* < .001, partial η^2^ = 0.06, and liked the reviewer for the High Status Organization (M = 56.52, SD = 20.34) more than the reviewer for the Low Status Organization (M = 52.83, SD = 21.26), *F*(1, 630) = 4.80, *p* = .029, partial η^2^ = 0.01. Participants thought that the Higher Class reviewer was more trustworthy (M = 62.54, SD = 21.26) than the Lower Class reviewer (M = 56.85, SD = 23.72), *F*(1, 630) = 10.25, *p* = .001, partial η^2^ = 0.02, and thought that the Higher Class reviewer had more knowledge about eyeglasses (M = 62.27, SD = 19.99) than the Lower Class reviewer (M = 53.80, SD = 22.40), *F*(1, 630) = 25.02, *p* < .001, partial η^2^ = 0.04. Each dimension of reviewer evaluation improved in relation to reviewer social class, suggesting that each dimension may play a role in explaining how reviewer class affects evaluations of the organization.

**A mediation model for testing whether changes in reviewer evaluations (DV2) could help explain changes in evaluations of the organization (DV1)**

**Fig 6:**
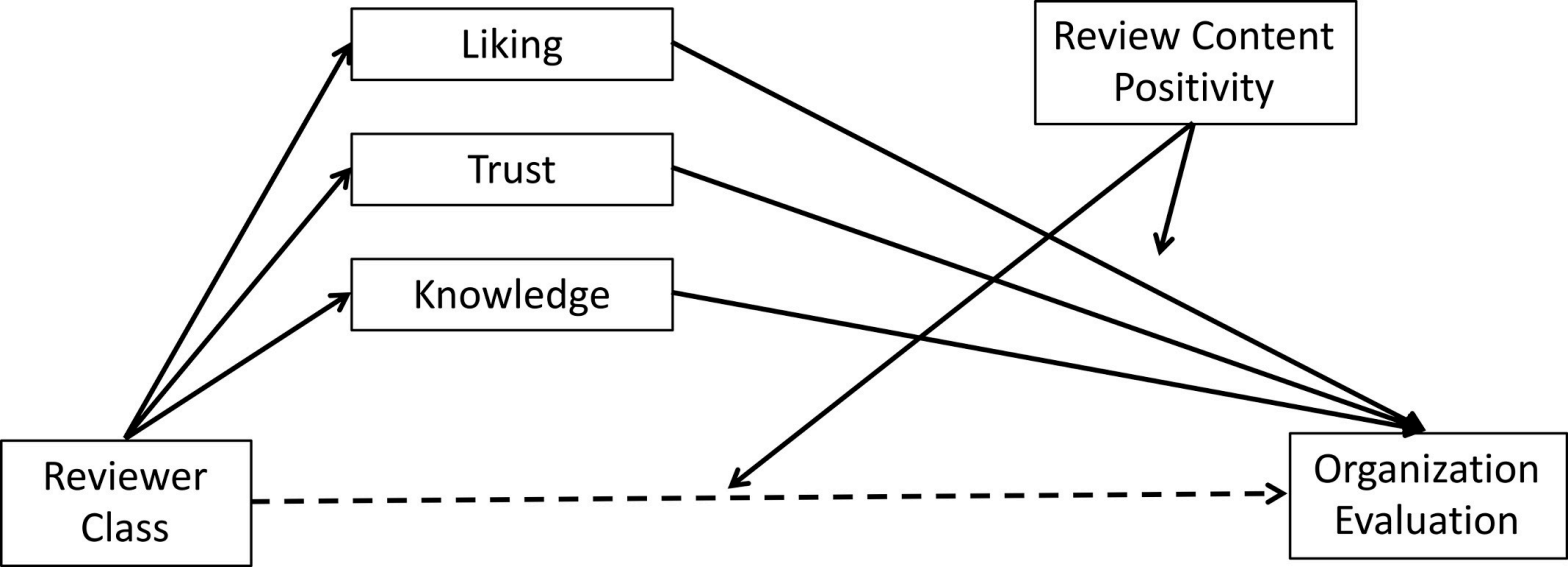
Schematic representation of the bootstrapping mediation analysis from Study 3, with Organization Status included as a covariate.

We tested whether changes in reviewer evaluations could help explain changes in evaluations of the organization. Specifically, we tested whether the effect of Reviewer Class on Organizational Evaluations was mediated by the three reviewer evaluations (Liking, Trust, and Knowledge), using a bootstrapping mediation (PROCESS model 15 [50]). We entered the three reviewer evaluations (variables mean-centered) as parallel mediators. We entered Review Positivity as a moderator of the relationship between Class and the three reviewer evaluations, and Organization Status was entered as a covariate (the results are robust to excluding this covariate). Fig 6 shows a schematic overview of the mediation model.

Results revealed that Reviewer Liking positively mediated the relationship between Reviewer Class both when the reviewer gave a Positive Review, 0.86 (0.47) 95% CI [0.16, 2.10], and when the reviewer gave a Negative Review, 0.90 (0.51) 95% CI [0.17, 2.32], again consistent with the “contagion” hypothesis. Because higher levels of reviewer liking lead to more positive organization evaluation in both conditions, there was no moderation of this effect by review content.

In contrast, reviewer Trust was a positive mediator when the review was Positive, 1.37 (0.57) 95% CI [0.48, 2.76], but was a negative mediator when the review was Negative, -2.32 (0.84) 95% CI [-4.27, -0.92]. The Trust variable demonstrated moderated mediation, indicated by a significant index of moderated mediation, 3.69 (1.30) 95% CI [1.45, 6.52], because increased reviewer trust lead to more positive organization evaluations when the review content was positive but increased trust lead to less positive evaluations when the review content was negative. This pattern is consistent with Hypothesis 2, that people generally adopt the opinions of those with higher-social class. Reviewer Knowledge was not a significant mediator in either the Positive, 0.62 (0.50) 95% CI [-0.21, 1.78] or Negative review condition, 0.14 (0.59) 95% CI [-1.05, 1.30]. We note, however, that when Reviewer Knowledge was the only variable entered as a mediator, it was a significant mediator. This helps reconcile this finding with the findings of Study 2.

#### Do the effects vary by the social class of participants?

Additionally, we examined the role of participants’ own social class in shaping their responses to our experimental conditions. We analyzed Organizational Liking scores with an ANOVA that included both the experimental variables and participants’ self-reported social class (categories coded 1–5). As seen in the primary analyses, there were main effects of Review Content, *F*(1, 601) = 110.93, *p* < .001, and Organization Status, *F*(1, 601) = 8.95, *p* = .003, and an interaction between Reviewer Class and Review Content, *F*(1, 601) = 7.08, *p* = .008. There was neither a main effect of participant class nor any interaction effects including participants class, all *p*s > .10.

### Discussion

Study 3 replicates the previous findings that endorsements from reviewers who are relatively higher in social class compared to reviewers with lower social class lead to more positive evaluations of the organization, regardless of whether the organization is higher or lower in status. Yet, when the reviewer offered a negative review of the organization, we observed a different pattern of results: having a higher-class reviewer did not improve organizational evaluations. Although the mean organizational evaluation was lower when the higher-class reviewer offered a negative review compared to when the lower-class reviewer offered a negative review, this mean difference did not reach statistical significance.

At first, these results do not fit cleanly with neither Hypothesis 1 *(the “Contagion” hypothesis)* nor Hypothesis 2 *(the “Higher class expertise” hypothesis)*, taken separately. The combination of these two effects, however, may explain the observed results. In the positive review conditions both processes lead to enhanced organizational evaluations for higher class reviewers, and therefore the two effects reinforce each other, leading to a positive and significant effect. In the negative review conditions, however these two processes counteract each other (the Contagion mechanism causes a positive effect, while the Higher-class expertise causes a negative effect). These two paths, therefore, may cancel each other out, leading to the observed non-significant results.

Examining reviewer evaluations as mediators of the effect of reviewer class on organizational evaluations provides additional support for the idea that both contagion processes and knowledge processes are occurring: ratings of reviewer liking have a positive mediation effect in both the positive and negative review condition, suggesting that contagion processes occur even when the reviewer says something negative about the organization, in line with Hypothesis 1 (the “Contagion” Hypothesis). But, ratings of reviewer trust positively mediate organization evaluations in the positive review condition and negatively mediate organization evaluations in the negative review condition, suggesting that the opinions of higher-class reviewers have more impact, although not due changes in perceived trustworthiness not perceptions of specific knowledge.

We note that in this study, we did not find support for Hypotheses 3, i.e., that higher-class reviewers were most influential when reviewing higher-priced organizations. Neither did we find support for Hypothesis 4, that is, we did not find a significant interaction between the experimental participant’s self-reported social class and the weight the participant puts on reviews on lower vs. higher-class reviewers. One reason for these non-findings may be that we did not have much variance in terms of the social class of the participants. Other reasons may include that our manipulations may not have been strong enough to detect such patterns; or maybe that our predictions are less likely to hold in the eye-glasses domain where people are buying a product, while it would maybe apply more in domains where quality is more elusive, such as in restaurants (note that we find evidence for these effects in Study 1, where most of the reviews are about restaurants). A third possible reason is that in Studies 2 and 3 participants self-report their social class, while in Study 1 we [the researchers] assess the reviewers’ social class based on their consumption patterns; prior research has demonstrated that these two approaches may lead to significantly different assessments of social class [51]. We call for future research to explore these possibilities.

## General discussion

Our research adds to growing body of work showing that social class shapes the way people interact [52], and particularly extends the current literature on interclass interactions to include responses to online reviewers [49]. Across one observational field study and two experiments, we demonstrate that reviewers’ social class backgrounds influence the ways in which potential customers respond to online reviews about organizations. When higher-class reviewers endorse an organization, they can enhance people’s evaluations of the organization, more so than when a lower-class reviewer offers the same endorsement. However, when higher-class reviewers disapprove of an organization, they do not necessarily have a stronger negative impact. This pattern of results is best explained by two processes co-occurring–positivity associated with the higher-class reviewer spreads to the organization via “contagion,” (see Hypothesis 1) and potential customers have increased trust in the (sometimes negative) opinions of higher-class reviewers (Hypothesis 2). These patterns highlight the crucial moderating effect of the content of the review: when the reviews is positive, the effects of “contagion” and “higher-class influence” reinforce each other, while when the review is negative the two effects cancel out each other (the contagion effect leads to a positive effect of reviewer status on evaluation, while the higher-class influence effect predicts a negative effect).

The findings are more mixed regarding Hypotheses 3 and 4. In our field study, we found evidence for Hypothesis 3 *(“Organization-reviewer status matching” hypothesis)*, and found that that higher-class reviewers were most influential when reviewing higher-priced organizations. In contrast, in the experimental studies we did not observe any interactions between the reviewers’ manipulated class and the manipulated organization status These differences could be due to several factors. One possibility is that the field study included many different product domains, while our experiments focused on only two product domains. Future research could systematically investigate whether the status of an organization, and the class of a review-reader, matter more in some domains than others. Previous research has shown that in the music domain people are sensitive to the class status of a music genre [53], suggesting that the class of reviewers writing about music might matter even more than in the domains examined here.

Regarding Hypothesis 4 (the “Reviewer-participant status matching” hypothesis), the results are also mixed. In Study 1, we observed a significant interaction between participant class, reviewer class, and reviewer opinion, and found that for a lower-class reviewer, other lower-class reviewers’ opinions are more influential than opinions from higher-class reviewers; vice versa for a higher-class participant. We did not find evidence for this effect in the experimental studies. As mentioned, one reason for these non-findings may be that the experiment participants we recruited via MTurk do not have much variance in terms of social class. Other reasons may include that our manipulations may not have been strong enough to produce detectable, significant patterns.

The inconsistent results may also be simply due to possible difference between a naturalistic setting of the online Yelp reviews and the more artificial experimental setting. Maybe online review writers take the task more seriously, therefore the effects we proposed are operating more strongly in the naturalistic setting. It may also be that the case that the results are stronger in the naturalistic setting because online reviewers select into reviewing domains and organizations that they care about, while experimental participants may care less about the organization they are presented with (because they have no choice). Future research may extend our experimental paradigm by giving a choice to participants regarding what kind of organizations or products they would like to read about.

It may also be the case that these factors matter more at other points in the decision-making process. In the experiments, our participants were given one specific review and asked to evaluate the organization, but this experimentally-guided decision-making process may differ significantly from the way that people naturally use online reviews. Previous research shows that people were more likely to read online posts written by men than posts written by women, but did not evaluate the content of the posts in a gender-biased way [5]. A similar multi-step process may occur in the domain of consumer opinions, where people might choose to read reviews from matched-status reviewers, but do not respond differently to a “matched-class” reviewer when given a single review to read. Future studies could explore the many decisions that people make when they are seeking information about an organization, such as which reviews to read in detail and when to stop gathering information about an organization. An experimental design that presented participants with conflicting reviews from multiple reviewers with different class background could also increase the realism of the paradigm.

Similarly, the participant samples in the observational study and the experimental studies may differ, and our experimental sample may not have included enough participants that identified with the perceived class of the fictional reviewers (e.g., participants may see themselves as middle-class rather than either lower- or higher-class). More broadly, our samples are limited because they are limited to the United States population or people who visit the United States (the observational study includes only reviews of US organizations, and the online experiment participants were limited to those in the US). These samples are neither specifically representative of the population that uses online reviews (as the Yelp dataset only includes those who write reviews, not all users), nor the United States as a whole, nor the general human population. Extending this research to other populations, perhaps from cultures with different views toward social class and materialism, could help illuminate the extent to which these patterns of responding rely on culture-specific beliefs about class (i.e., American beliefs about meritocracy).

Future research could build upon our experimental design in other ways as well. First, the reviewers pictured were always male, and some research suggests that social class biases may function differently in evaluations of men versus women [54, 55]. It may be fruitful to examine how class and gender (and other social identity characteristics such as age, or race) intersect in this domain. Second, we manipulated the reviewers’ social class to be relatively higher and lower in social class (73 and 35, respectively, on a scale of 100 in Experiment 1; 69 and 32 in Experiment 2), but it may be fruitful to probe responses to wider range of social classes. For example, an “Ultra Rich” reviewer (i.e., someone rating 95 on a scale of 100) may be treated differently than the moderately Higher Class reviewer portrayed in these studies, as the “rich” are often stereotyped as dishonest [56], and a dishonest opinion may be disregarded.

Future research could also investigate further the circumstances under which online review users rely on peripheral cues in general, and on other reviewers’ social class in specific. The Elaboration Likelihood Model of Petty and Cacioppo [3] argues that the less important the topic is for a decision maker, the less knowledge they have about the topic, or the less time they have to make a decision, the more likely they will rely on peripheral cues in making decisions. Translating this insight into the setting of our study, we expect that these factors would also affect the extent to which online review readers would rely on social class information as a cue.

Our research also fits within a larger literature examining different types of social class evaluations, and the extent to which social class evaluations result from conscious versus non-conscious processing. The two processes supported by our findings, a “contagion” process and a “trust in expertise” process, may result from different types of social class evaluations. Previous research has found that people consciously endorse *stereotypes* of the higher class as highly competent but low in warmth, and the lower class as low in both competence and warmth [57]. If explicit stereotypes of the higher class as experts are driving our results, this process may occur within people’s awareness. Yet, other research on social class *attitudes* has shown that although people do not say that they like the higher-class and instead say that they prefer the middle-class, assessments of people’s implicit, non-conscious attitudes indicates that the rich are associated with positivity, even more so than the middle- or lower-classes [58]. Since positivity with higher class people is seen only at the implicit level, the “contagion” of positivity seen in our results may be a non-conscious process. Future research could examine these processes in more detail, perhaps by measuring participants’ social class attitudes and stereotypes and then probing the relationship between social class evaluations and responses to reviewers.

Overall, we hope that our research examining responses to higher- and lower-class reviewers adds to both a growing literature on the psychology of class relations and to an understanding of online review usage. We have uncovered some interesting mechanisms but some of our predictions have received mixed results across the observational and the experimental settings. Taken together, the results indicated that the social class processes behind online reviewing result from multiple, often reinforcing but often contradictory, psychological processes. Our paper is the first in starting to examine these processes, but clearly much more work remains to be done to fully understand the complex nature of the effects of social class on online reviewing.
